# 

**Published:** 1902-10

**Authors:** 


### BOOKS RECEIVED.

Saunders's Question Compends. Essentials of Histology. By Louis Leroy, B. S., M. D., Professor of Histology and Pathology, Vanderbilt University, Medical and Dental Departments; Pathologist to the Nashville City Hospital, etc. Second edition, thoroughly revised and greatly enlarged. 16mo volume of 263 pages, with 92 beautiful illustrations. Philadelphia and London: W. B. Saunders & Co. 1902. [Cloth, $1.00 net.]

Saunders's Question Compends. Essentials of Diseases of the Ear. By E. B. Gleason, S. B., M. D., Clinical Professor of Otology, Medico-Chirurgical College, Philadelphia; Surgeon in charge of the Nose, Throat and Ear Department of the Northern Dispensary, Philadelphia, etc. Third edition, thoroughly revised. 16mo volume of 214 pages, with 114 illustrations. Philadelphia and London: W. B. Saunders & Co. 1902. [Cloth, $1.00 net.]

Practical Diagnosis. The Use of Symptoms and Physical Signs in the Diagnosis of Disease. By Hobart Amory Hare, M. D., B. Sc., Professor of Therapeutics in the Jefferson Medical College of Philadelphia; Physician to the Jefferson Medical College Hospital; Author of a Textbook of Practical Therapeutics. Fifth edition, revised and enlarged. Octavo, pp. 710. Illustrated. Philadelphia and New York: Lea Brothers & Co. 1902. [Price, cloth, $5.00 net.]

General Paresis, Practical and Clinical. By Robert Howland Chase, A. M., M. D., Physician-in-Chief, Friends Asylum for the Insane, Philadelphia; Late Resident Physician, State Hospital, Norristown, Pa. Duodecimo, 290 pages. Illustrated. Philadelphia: P. Blakiston's Son & Co. 1902. [Price, $1.75 net.]

The Practical Medicine Series of Year Books. Comprising ten volumes on the year's Progress in Medicine and Surgery. Issued monthly under the general editorial charge of Gustavus P. Head, M. D., Professor of Laryngology and Rhinology in the Chicago Post-Graduate Medical School. Volume IX. Physiology, Pathology, Bacteriology, Anatomy. Pathology edited by W. A. Evans, M. S., M. D., Professor of Pathology College of Physicians and Surgeons, Chicago. Bacteriology, edited by Adolph Gehrman, M. D., Professor of Bacteriology, College of Physicians and Surgeons, Chicago. Chicago: The Year Book Publishers. 1902. [Price, $1.25; entire series, $7.50]

A Treatise on Diseases of the Anus, Rectum and Pelvic Colon. By James P. Tuttle, A. M., M. D., Professor of Rectal Surgery in the New York Polyclinic Medical School and Hospital. Octavo, pp. 980. Illustrated. New York: D. Appleton & Co. 1902. [Price, cloth, $6.00; half-leather, $6.50.]

The Diseases of Infancy and Childhood. For the Use of Students and Practitioners of Medicine. By L. Emmett Holt, M. D., LL. D., Professor of Diseases of Children in the College of Physicians and Surgeons (Columbia University), New York; attending Physician to the Babies' and Foundling Hospitals, Octavo, pp. 1178. Illustrated. Second edition, revised and enlarged. New York: D. Appleton & Co. 1902. [Price, cloth, $6.00; half-leather, $6.50.]

The Diseases of Infancy and Childhood. Designed for the use of Students and Practitioners of Medicine. By Henry Koplik, M. D., Attending Physician to Mt. Sinai Hospital, New York. Octavo, pp. 675. Illustrated with 169 engravings and 30 plates in color and monochrome. Philadelphia and New York: Lea Brothers & Co. 1902. [Price, cloth, $5.00 net.]

Blakiston's Quiz Compends. Compend of Special Pathology. By Alfred Edward Thayer, M. D., Assistant Instructor in Gross Pathology, Cornell Medical College; Pathologist to the City Hospital, New York. Duodecimo, pp 322. Illustrated. Philadelphia. P. Blakiston's Son & Co. 1902. [Price, 80 cents net.]

Diseases of the Rectum and Anus. Designed for Students and Practitioners of Medicine. By Samuel Goodwin Gant, M. D , LL. D., Professor of Rectal and Anal Surgery at the New York Post-Graduate Medical School and Hospital; formerly Professor of Gastrointestinal Surgery at the University and Woman's Medical Colleges, Kansas City, Mo.; Attending Surgeon for Rectal and Anal Diseases to the New York Post-Graduate Hospital, St. Mark's Hospital, Hebrew Sheltering Guardian Orphan Asylum and New York Infant Asylum, etc. Second edition, rewritten and enlarged with 37 full-page plates, 20 of which are in colors and 212 smaller engravings and half-tones. Pages xxiv.-687. Royal octavo. Philadelphia: F. A. Davis Company. 1902. [Extra cloth, $5.00 net; sheep or half-Russia, $6.00 net, delivered.]

A Treatise on the Principles and Practice of Gynecology. By E. C. Dudley, A. M., M. D., Professor of Gynecology in the Northwestern University Medical School, Chicago. New (3d) edition, enlarged and thoroughly revised. In one very handsome octavo volume of 756 pages, with 474 engravings, of which 60 are in colors and 22 colored plates. Philadelphia and New York: Lea Brothers & Co. 1902. [Cloth, $5.00 net; leather, $6.co net; half morocco, $6.50 net.]

Diseases of the Stomach. Their Special Pathology, Diagnosis and Treatment, with sections on Anatomy, Physiology, Chemical and Microscopical Examination of Stomach Contents, Dietetics, Surgery of the Stomach, etc. By John C. Hemmeter, M. D., Philos. D., Professor in the Medical Department of the University of Maryland, Baltimore, etc. Third revised and enlarged edition. Octavo, pp. 883. Illustrated. Philadelphia: P. Blakiston's Son & Co. 1902. [Price, $6.00 net.]

Transactions of the American Dermatological Association. Twenty-fifth Annual Meeting held at Chicago, May 30 and 31 and June 1, 1901. Frank Hugh Montgomery, M. D., Secretary. New York: Rooney & Otten Printing Co. 1902.

The International Textbook of Surgery. In two volumes. By American and British Authors. Edited by J. Collins Warren, M. D., LL. D., F. R. C. S. (Hon.), Professor of Surgery, Harvard Medical School; and A. Pearce Gould, M. S., F. R. C. S., of London, England. Second edition, thoroughly revised and enlarged. Vol. I. General and Operative Surgery. Royal octavo of 965 pages, with 461 illustrations and 9 full-paged colored lithographic plates. Vol II. Special or Regional Surgery. Royal octavo of 1,122 pages, with 499 illustrations, and 8 full-paged colored lithographic plates. Philadelphia and London: W. B Saunders & Co. 1902. [Cloth, $5.00 net; sheep or half-morocco, $6.00 net.]

The Treatment of Fractures. By Chas. L. Scudder, M. D., Assistant in Clinical and Operative Surgery, Harvard Medical School. Third edition, revised and enlarged. Octavo, 480 pages, with 645 original illustrations. Philadelphia and London: W. B. Saunders & Co. 1902. [Polished buckram, $4.50 net; half-morocco, $5.50 net.]

A Textbook of Materia Medica, Therapeutics and Pharmacology. By George F. Butler, Ph. G., M. D., Professor of Materia Medica and Therapeutics in the College of Physicians and Surgeons, Chicago, Medical Department of the University of Illinois, etc. Fourth edition, thoroughly revised. Handsome octavo volume of 896 pages, illustrated. Philadelphia and London: W. B. Saunders & Co. 1902. [Cloth, $4.00 net; sheep or half-morocco, $5.00 net.]

A Textbook of the Surgical Principles and Surgical Diseases of the Face, Mouth and Jaws. For Dental Students. By H. Horace Grant, A. M., M. D., Professor of Surgery and of Clinical Surgery, Hospital College of Medicine; Professor of Oral Surgery, Louisville College of Dentistry, Louisville. Octavo volume of 231 pages, with 68 illustrations. Philadelphia and London: W. B. Saunders & Co. 1902. [Cloth, $2.50 net.]

Saunders's Medical Hand-Atlases. Atlas and Epitomé of Traumatic Fractures and Dislocations. By Professor Dr. H. Helferich, Professor of Surgery at the Royal University, Greifswald, Prussia. Edited, with additions, by Joseph C. Bloodgood, M. D., Associate in Surgery, Johns Hopkins University, Baltimore.

From the fifth revised and enlarged German edition. With 216 colored illustrations on 64 lithographic plates, 190 text-cuts, and 353 pages of text. Philadelphia and London: W. B. Saunders & Co. 1902. [Cloth, $3.00 net.]

Practical Obstetrics. A Textbook for Practitioners and Students. By Edward Reynolds, M. D., Visiting Surgeon to the Free Hospital for Women; and Franklin S. Newell, M. D., Assistant in Obstetrics and Gynecology in Harvard University. Octavo, pp. 553. Illustrated with 252 engravings and three colored plates. Philadelphia and New York: Lea Brothers & Co. 1902. [Price, $3.75 ]

Handbook of Physiology. Revised by William H. Rockwell, Jr., M. D., and Charles L. Dana, A. M., M. D., Professor of Diseases of the Nervous System, Cornell University Medical College, New York; Physician to Bellevue Hospital. Octavo, pp. 865. Seventeenth American edition. With upwards of 500 illustrations, many in colors. New York: William Wood & Co. 1902. [Price, $3.co net.]

The Practical Medicine Series of Year Books. Ten volumes. Issued monthly, under the general editorial charge of Gustavus P. Head, M. D., Professor of Laryngology and Rhinology in the Chicago Post-Graduate Medical School. Volume X. Skin and Veneral Diseases, Nervous and Mental Diseases. Edited by W. L Baum, M. D., Hugh S. Patrick, M. D. Duodecimo, pp. 245. Chicago: The Year Book Publishers. 1902. [Price, $1.25; entire series, $7.50.]

Transactions of the Medical Society of the State of New York. Ninety-sixth Annual Meeting held at Albany, January 28, 29 and 30, 1902. Frederic C. Curtis, M. D., Secretary. Published by the Society. 1902.

Progressive Medicine. Volume III, September, 1902. A Quarterly Digest of Advances, Discoveries and Improvements in the Medical and Surgical Sciences. Edited by Hobart Amory Hare, M D., Professor of Therapeutics and Materia Medica in the Jefferson Medical College of Philadelphia. Philadelphia and New York: Lea Brothers & Co. [Per annum, in four cloth-bound volumes, $10.00. Per volume, $2.50, by express prepaid to any address.]

Physical Diagnosis. Diseases of the Thoracic and Abdominal Organs. A Manual for Students and Physicians. By Egbert LeFevre, M. D., Professor of Clinical Medicine and Associate Professor of Therapeutics in the University and Bellevue Hospital Medical College. Illustrated. Philadelphia: Lea Brothers & Co. 1902.

A Textbook of Histology and Microscopic Anatomy of the Human Body. Including Microscopic Technique. By Dr. Ladislaus Szymonowicz, A. Ö., Professor of Histology and Embryology in the University of Lemberg. Translated and edited by John Bruce MacCallum, M. D., Johns Hopkins University, Baltimore. Octavo, pp. 435. Illustrated with 277 engravings, including 57 plates in colors and monochrome. Philadelphia and New York: Lea Brothers & Co. 1902. [Price, cloth, $4.75 net.]

Lea's Series of Pocket Textbooks. Materia Medica, Therapeutics, Medical Pharmacy, Prescription Writing and Medical Latin. A Manual for Students and Practitioners. By William Schleif, Ph. G., M. D., Instructor in Pharmacy, University of Pennsylvania. Series edited by Bern B. Gallaudet, M. D., Demonstrator of Anatomy and Instructor in Surgery, College of Physicians and Surgeons, New York. Duodecimo, pp. 389 Second edition, revised and enlarged. Philadelphia and New York: Lea Brothers & Co. 1902. [Price, cloth, $1.75.]

